# Vaporization of perfluorocarbon attenuates sea-water-drowning-induced acute lung injury by deactivating the NLRP3 inflammasomes in canines

**DOI:** 10.3389/ebm.2024.10104

**Published:** 2024-04-19

**Authors:** Cheng-Cheng Su, Zhao-Rui Zhang, Jin-Xia Liu, Ji-Guang Meng, Xiu-Qing Ma, Zhen-Fei Mo, Jia-Bo Ren, Zhi-Xin Liang, Zhen Yang, Chun-Sun Li, Liang-An Chen

**Affiliations:** ^1^ Medical School of Chinese PLA, Beijing, China; ^2^ Department of Respiration, The Eight Medical Center of Chinese PLA General Hospital, Beijing, China; ^3^ Department of Critical Care and Respiration, Characteristic Medical Center of Chinese People’s Armed Police Force, Tianjin, China

**Keywords:** perfluorocarbons, acute lung injury, probe-based confocal laser endomicroscopy, heme oxygenase-1, nuclear respiratory factor-1

## Abstract

Seawater-drowning-induced acute lung injury (SD-ALI) is a life-threatening disorder characterized by increased alveolar–capillary permeability, an excessive inflammatory response, and refractory hypoxemia. Perfluorocarbons (PFCs) are biocompatible compounds that are chemically and biologically inert and lack toxicity as oxygen carriers, which could reduce lung injury *in vitro* and *in vivo*. The aim of our study was to explore whether the vaporization of PFCs could reduce the severity of SD-ALI in canines and investigate the underlying mechanisms. Eighteen beagle dogs were randomly divided into three groups: the seawater drowning (SW), perfluorocarbon (PFC), and control groups. The dogs in the SW group were intratracheally administered seawater to establish the animal model. The dogs in the PFC group were treated with vaporized PFCs. Probe-based confocal laser endomicroscopy (pCLE) was performed at 3 h. The blood gas, volume air index (VAI), pathological changes, and wet-to-dry (W/D) lung tissue ratios were assessed. The expression of heme oxygenase-1 (HO-1), nuclear respiratory factor-1 (NRF1), and NOD-like receptor family pyrin domain containing-3 (NLRP3) inflammasomes was determined by means of quantitative real-time polymerase chain reaction (qRT-PCR) and immunological histological chemistry. The SW group showed higher lung injury scores and W/D ratios, and lower VAI compared to the control group, and treatment with PFCs could reverse the change of lung injury score, W/D ratio and VAI. PFCs deactivated NLRP3 inflammasomes and reduced the release of caspase-1, interleukin-1β (IL-1β), and interleukin-18 (IL-18) by enhancing the expression of HO-1 and NRF1. Our results suggest that the vaporization of PFCs could attenuate SD-ALI by deactivating NLRP3 inflammasomes via the HO-1/NRF1 pathway.

## Impact statement

The study offered the first pCLE image description and quantitative analysis of acute lung injury induced by seawater drowning, and demonstrated that inhalation of vaporized PFCs suppressed seawater-drowning-induced acute lung injuries. The mechanisms of PFCs’ therapeutic effects may be attributed to the regulation of NLRP3 inflammasomes’ activation via the HO-1/NRF1 pathway. This study offers a meaningful exploration of PFCs’ therapeutic mechanisms *in vivo*.

## Highlights

• We observed that PFCs can attenuate seawater-drowning-induced acute lung injury.
• In this study, we proved that probe-based confocal laser endomicroscopy is a noninvasive examination to evaluate acute lung injury realtimely.
• Regulation of NLRP3 inflammasomes is a new promising target for seawater-drowning-induced acute lung injury.
• HO-1/NRF1 pathway connects with NLRP3 activation.

## Introduction

Seawater drowning is a major cause of morbidity and mortality worldwide. According to the World Health Organization, drowning accounted for an estimated 372,000 deaths in 2012 [1]. Seawater-drowning-induced lung injury (SD-ALI)/acute respiratory distress syndrome (SD-ARDS) is one of the most important complications in drowning patients, with a high fatality rate but few therapies [2, 3]. The main treatment for drowning is supportive care, including pulmonary support to avoid complications. Previous studies have suggested that treatments including dexamethasone, tanshinone II A and 1α, and 25-dihydroxyvitamin D3 might be effective at alleviating SD-ALI [4–7]. However, we still lack an effective therapy for this life-threatening disorder. ALI/ARDS are often regarded as one entity with similar pathobiology of lung injuries, clinical manifestations, and specific targets of drug intervention, despite induced by different injuries and conditions. The category “acute lung injury”, the previous definition expressed the milder form of ARDS, was omitted from the Berlin definition. It is generally considered that this term is the description of the situation where the clinical definition criteria cannot be fulfilled as well as experimental study [8]. According to current research, SD-ALI is generally recognized as a complex injury process involving an insufficiency of oxygen and pulmonary surfactant, blood–air barrier disruption, the formation of pulmonary oedema, inflammation, oxidative stress, autophagy, apoptosis, and so on, but its exact mechanisms and potential treatments require further exploration [9].

Perfluorocarbons (PFCs) are colorless and odorless molecular liquids composed of carbon and fluorine with features such as a high level of gas dissolvability in water, rapid release, low surface pressure, high volume, average volatility, favorable histo-compatibility, and no absorption or metabolism *in vivo* [7, 10–12]. Partial liquid ventilation with perfluorocarbons improves the gas exchange, breathing mechanism, and lung structure in many acute lung injury models [13–16]. Our previous studies showed that blast lung injuries could be attenuated by the vaporization of PFCs via the nuclear factor (NF)-κB pathway *in vivo* and *in vitro* [17, 18]. However, the influence of PFCs on SD-ALI in canines has not been reported.

Probe-based confocal laser endomicroscopy (pCLE) is a minimally invasive technique that allows for the *in vivo* microscopic imaging of living tissue through a 1 mm flexible fiber optic mini probe [19]. Recent studies showed that pCLE has the potential to serve as an *in vivo* bronchoscopic imaging technique in the assessment of lung injury [20]. Our study aimed to explore whether PFCs could reduce the severity of SD-ALI and investigate the underlying molecular mechanisms.

## Materials and methods

### Chemicals and reagents

The experimental seawater was prepared according to the formula provided by the Third Institute of Oceanography, Ministry of Natural Resources, Fujian, China. The main components of the seawater were close to those of the southeast coastal seawater of China: pH, 8.2; specific gravity, 1.05; and osmotic pressure, 1,300 mmol/L; NaCl 26.518 g/L, MgCl_2_ 2.447 g/L, MgSO_4_ 3.305 g/L, CaCl_2_ 1.141 g/L, KCl 0.725 g/L, NaHCO_3_ 0.202 g/L, NaBr 0.725 g/L [21].

### Animal experiment and modeling

Eighteen male specified pathogen-free beagle dogs (12–18 months old, weighing 13.02 ± 0.66 kg) were obtained from the Laboratory Animal Center of the PLA General hospital. Dogs were housed under a 12 h light/dark cycle in a room with a controlled temperature (23°C) and humidity (50%). All experiments were performed in compliance with the Experimental Animal Care Committee of the PLA General hospital (SQ2022447) and the ARRIVE 2.0 guidelines [22].

We randomly divided the 18 beagle dogs into 3 groups: the control, seawater-induced acute lung injury (SW), and PFC groups. Anesthesia was induced via intraperitoneal (I.P.) injection of pentobarbital (25–30 mg/kg), which was deemed adequate according to the blink test throughout the experiment. Following 15 min of stabilization, the dogs in the control group were subjected to tracheal intubation without seawater instillation and treated with a ventilator (DRAGER, Savina 300) according to the following parameters: 30% inspired oxygen (FiO_2_), 8 mL/kg tidal volume (VT), 4 cmH_2_O positive end-expiratory pressure (PEEP), and 16 counts/min. The dogs in the SW groups were subjected to tracheal intubation and instilled with pre-cooled seawater (8 mL/kg) through a tracheal cannula within 1 min. The dogs were maintained in a supine position with heads elevated at 30° for monitoring. The appearance of dyspnea and frothy sputum, as well as a sharp drop in the oxygen saturation to 45–65%, demonstrated the successful modeling of SD-ALI. After resuscitation, the dogs were treated with a ventilator according to the same parameters as the control group. The dogs in the PFC groups were subjected to tracheal intubation and instilled with pre-cooled seawater (8 mL/kg), as described above in the SW group; then, they were ventilated with the same parameters, and vaporized PFCs (10 mL/kg) (C8F18; Huajieshi Medical Company, Shanghai, China)were administered continuously for 1.5 h at 0 h and 3 h after modeling via mechanical ventilation.

### Blood gas analysis

Arterial blood was collected from the femoral artery of the dogs using a blood gas needle after 0 h, 3 h, and 6 h of modeling. Then, blood gas was analyzed immediately using an automated blood gas analyzer (ABL 800, Radiometer, Copenhagen, Denmark).

### Probe-based confocal laser endomicroscopy

PCLE was performed with a 1.4 mm semi-flexible confocal probe (Cellvizio, Mauna Kea Technologies, Paris, France), which can pass through a portable bronchoscope. A 1 mL volume of 1% sodium fluorescein was intravenously administered to obtain clear images 1 min before pCLE. We inserted the probe via the endotracheal route under bronchoscope control into a proximal segmental bronchiole of the left lobe, as described previously, and recorded the video sequences at least for at least 30 s using imaging software (Image cell 3.6.2; Mauna Kea Technologies, Paris, France). The volume air index (VAI) was calculated as described previously [23]. Briefly, images at end-expiration were selected for analysis and exported as single frames. Then, we developed a batch image processing method for NI Vision Assistant 8.2.1 (National Instruments, Austin, TX, United States) and obtained a histogram including the grey value distribution that could be analyzed and transferred to an Excel sheet (Microsoft Excel, 2003). Then, we calculated the mean grey value M) and standard deviation (SD) to define the integration limits, defining M−SD as the lower limit and M as the upper limit. The pixels between the lower and upper integration limits represented air-filled structures in the predefined slice. The VAI equaled the number of counted pixels divided by their total number, which represented the air content of several binary layers.

### Sample collection

Blood and tissue samples from the lower right lung were harvested for subsequent measurements. We cut the left lung of the dogs and injected it with 20 mL pre-cooled normal saline. Then, we collected the bronchoalveolar lavage fluid (BALF) by gently washing the right lung. The lavage was repeated 3 times, and the retrieval rate was over 70%. The supernatants of the BALF were collected by centrifugation at 1,000 rpm for 5 min. The cells were resuspended in saline and smeared on glass slide for hematoxylin and eosin (H&E) staining and cell counting as described previously [24]. The three different sites in the anterior lobe, middle lobe, and posterior lobe of the right lung were fixed in 4% paraformaldehyde at room temperature, embedded in paraffin, sectioned serially at 5 μm, and stained with H&E for immunofluorescence and immunohistochemistry analyses. The peripheral venous blood was collected with a blood collection tube containing ethylene diamine tetraacetic acid (EDTA).

### Measurement of lung wet-to-dry weight (W/D) ratio

To determine the severity of lung edema and water accumulation, we measured the W/D ratios. The middle lobe of the right lung was weighed immediately after sampling, and then the tissues were dried at 65°C until the weight was stabilized. The lung tissue W/D ratios were calculated as wet weight divided by dry weight.

### H&E staining

Lung tissue sections were stained for histological analysis with a H&E staining kit. Finally, the slides were scanned using a Nano-Zoomer S60 system (Hamamatsu Photonics, Hamamatsu City, Japan). All sections were reviewed and analyzed by two pathologists independently in a blinded manner. The scoring strategy followed a previously described method [25]. Briefly, two pathologists who were blind to the treatment of the animals examined all tissue sections (3 sections from each animal) and evaluated the hyaline membrane, interstitial edema, intra-alveolar hemorrhage, intra-alveolar edema, and neutrophil accumulation levels. These characteristics were subjectively scored from 0 to 3: 0 = normal, 1 = slight effect, 2 = moderate presence of the feature, and 3 = severe effect.

### Transmission electron microscopy (TEM)

Lung tissue samples with the size of millets were fixed in 4% glutaraldehyde at 4°C for 24 h and post-fixed in 1% osmic acid for 2 h. After dehydration with a gradient of ethanol and acetone, the samples were embedded in an epoxy resin. Finally, ultrathin sections were stained with 2% uranylacetate and lead citrate and observed using transmission electron microscopy (HT7800, Hitachi, Tokyo, Japan).

### Immunological histological chemistry

Lung tissue sections were incubated in 3% H_2_O_2_ to block endogenous peroxidase and placed in blocking buffer (C220702, Yangguangbio, Beijing, China) for 15 min, followed by staining using rabbit polyclonal primary antibodies for NOD-like receptor family pyrin domain containing 3 (NLRP3) (1:500; bs-10021R, Bioss, Beijing, China), nuclear respiratory factor-1 (NRF1) polyclonal antibodies (1:1000; MBS3204137, Mybiosource, San Diego, CA, United States) and heme oxygenase-1 (HO-1) antibodies (1:500; GTX13248, Genetex, San Antonio, TX, United States) overnight at 4°C. Then, the sections were washed three times in PBS and incubated with horseradish peroxidase (HRP)-conjugated goat anti-rabbit IgG (H+L) (GB23303, Solarbio, Wuhan, Hubei, China) and HRP-conjugated goat anti-mouse IgG (H+L) (GB23301, Solarbio, Wuhan, Hubei, China). The slides were incubated in DAB (AR1022, Bosterbio, Wuhan, Hubei, China), counterstained in Mayer’s hematoxylin solution, dehydrated, cleared in xylene, and placed in Depex mounting medium. Finally, the slides were scanned using a Nano-Zoomer S60 system.

### Immunofluorescence

Lung tissue sections were placed in blocking buffer for 15 min followed by staining using rabbit polyclonal antibodies for E-cadherin polyclonal antibodies (1:300; 20874-1-AP, protein-tech, Wuhan, Hubei, China) overnight at 4°C. Then, the sections were washed 3 times in PBS and incubated in goat anti-rabbit IgG/RBITC (SR134, Solarbio, Wuhan, Hubei, China), followed by washing 3 times in PBS and incubation in anti-fade medium containing 4′,6-diamidino-2′-phenylindole (DAPI). Finally, the slides were scanned using a Nano-Zoomer S60 system.

### Enzyme-linked immunosorbent assay (ELISA)

The BALF supernatant obtained using the method described in *Sample collection* section was centrifuged at 8,000 *g* for 20 min, and we detected the levels of pulmonary surfactant A (SP-A) and pulmonary surfactant B (SP-B) with ELISA kits (Shanghai Enzyme-linked Biotechnology company, Shanghai, China) according to the manufacturer’s protocol.

### Quantitative real-time PCR (qRT-PCR) analysis

Using TRIzol™ reagent (15596026, Invitrogen, Carlsbad, CA, United States), total RNA was extracted from lung tissues. Then, we obtained the cDNA using a reagent kit (9767, TaKaRaBio, Dalian, Liaoning, China). QRT-PCR was carried out using a KAPA SYBR^®^ FAST qPCR Master Mix kit (KK4601, R&D, Cape Town, South Africa) with the amplification conditions set as follows: 94°C for 30 s, followed by 40 cycles at 94°C for 5 s and 60°C for 34 s. Three technical replicates were performed for each cDNA sample. The β-actin gene was employed as the internal control. The primer sequences for the target genes are listed in Table 1.

**TABLE 1:** Primer sequences used in this study.

Primers	Sequences (5′-3′)	PCR products (bp)
NRF1	CAC​CAA​TGG​GAG​CAA​TTT​TT	141
	ACA​CCC​AGG​TCC​CTT​TCT​CT	
HO-1	CTT​CTT​CAC​CTT​CCC​CAA​CA	138
	ATG​TTC​AGC​AGG​AAG​GCA​GT	
NLRP3	GTG​TTA​TGT​GGC​CCT​GGA​CT	119
	TGG​TGC​TTC​TGG​GTC​TCT​CT	
caspase-1	GCC​TCA​ACC​TCA​AGG​ACA​AA	101
	TGT​CGA​GGC​TTT​TGG​AGA​GT	
IL-1β	CAA​GAA​ACC​CTC​CTT​TGT​GC	142
	ACA​AGT​GGG​CTT​TTT​CAT​GG	
IL-18	ACG​AGG​GAA​ATC​AAC​CTG​TAT​T	113
	CAG​ACC​TCT​AGT​GAG​GCT​ATC​T D	
β-actin	TGC​GTG​ACA​TCA​AGG​AGA​AG	175
	AGG​AAG​GAA​GGC​TGG​AAG​AG	

Abbreviations used are follows: NRF1, nuclear respiratory factor-1; HO-1, heme oxygenase-1; NLRP3, NOD-like-receptor family pyrin domain-containing-3; IL-1β, interleukin-1β; IL-18, interleukin-18.

### Statistical analysis

Statistical analysis was performed using GraphPad Prism 6 software (GraphPad Software Inc., La Jolla, CA, United States). Data are presented as mean ± standard error of mean (SEM), and one-way analysis of variance followed by Tukey’s test was used for comparisons. *p* < 0.05 was considered to indicate a statistically significant difference.

## Results

### Blood gas analysis

**FIGURE 1:**
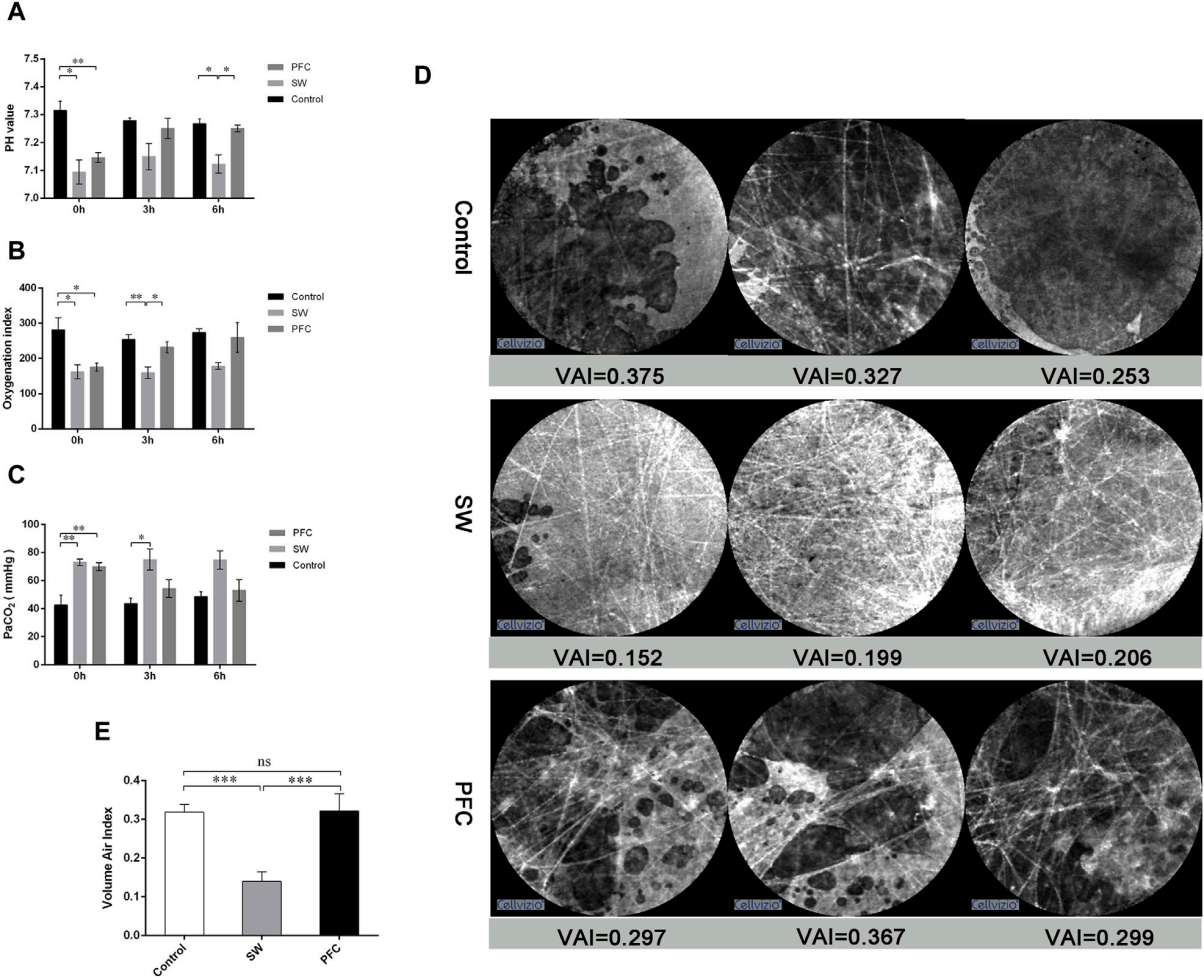
PFCs improved hypoxia and barrier damage induced by seawater. **(A)** pH values; **(B)** oxygenation index; **(C)** partial pressure of carbon dioxide at 0 h, 3 h, and 6 h after modeling. **(D)** Representative images from probe-based confocal laser endomicroscopy. **(E)** The volume air index. Data are presented as mean ± SEM. n = 6. * *p* < 0.05, ** *p* < 0.01, *** *p* < 0.001. Abbreviations: SW, seawater; PFC, perfluorocarbon, VAI, volume air index.

Compared with the control group, the pH value and oxygen index were significantly decreased and the PaCO_2_ significantly increased in the SW group (*p* < 0.05). The blood gas analysis for the PFC group showed a significantly increased PaO_2_ and pH value and decreased oxygen index (*p* < 0.05) (Figures 1A–C).

### PCLE helped to assess lung injury in a noninvasive and real-time manner

Images in the control group showed the fiber-like structures represented normal alveolar tissue, while the dark background represented the gas in the lung. However, the pictures from the SW group showed much thicker fiber-like structures and more fiber-like structures representing the thickening and disorder of alveolar structures, while the background became much thicker, representing the gas-trapping, liquid leakage and inflammatory infiltration. However, images from the PFC group showed alleviation of the above-mentioned manifestations (Figure 1D).

Then, image sequences of at least six differing alveolar clusters of each group were selected for the calculation of the VAI. As depicted in Figure 1E significant difference between the control group and the SW group was observed (*p* < 0.05), and PFC administration could significantly upregulate VAI compared with the SW group.

### Pathological changes

**FIGURE 2:**
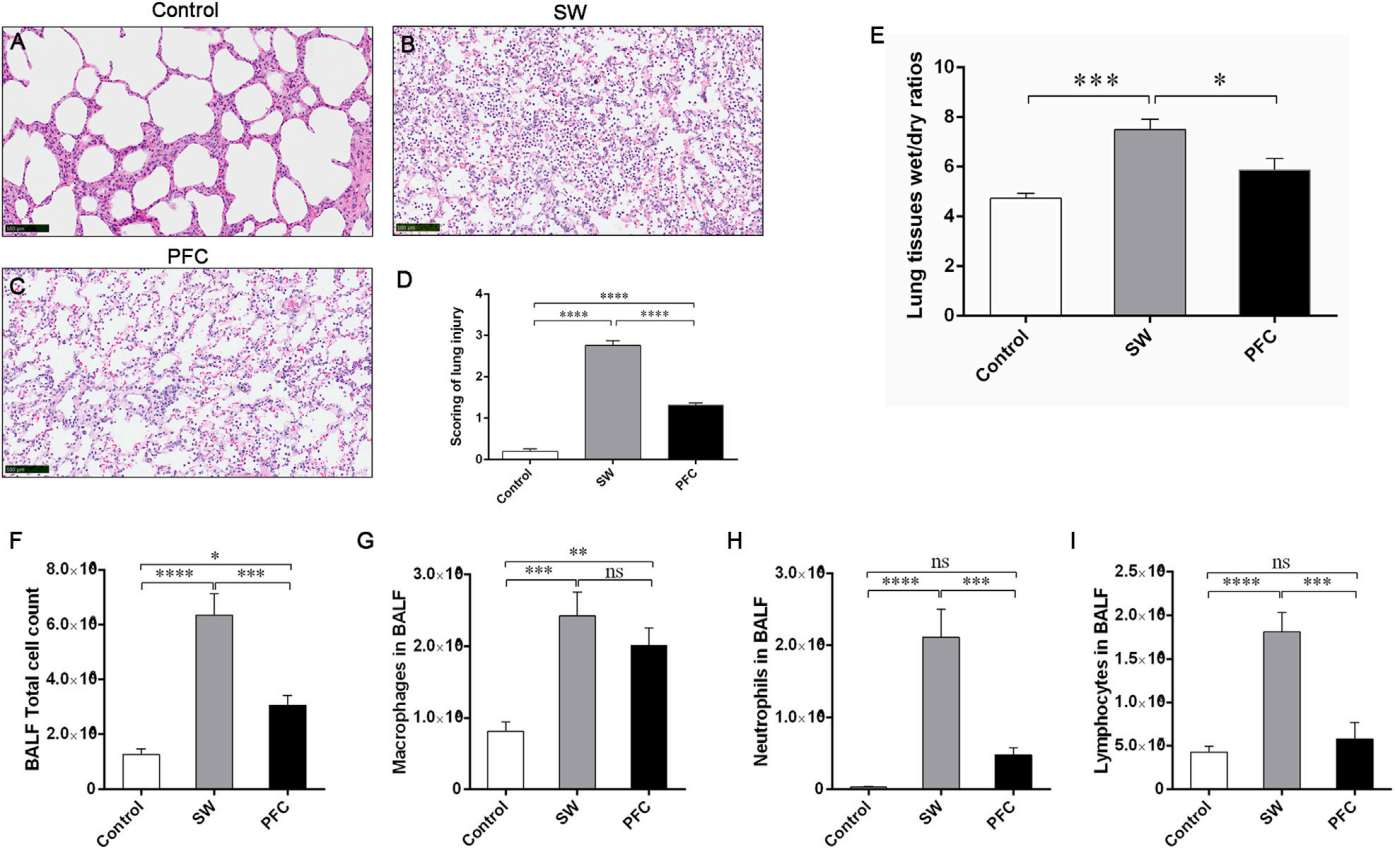
PFCs alleviated inflammatory infiltration and lung edema induced by seawater. The H&E staining of lung tissue from the control group **(A)** SW group **(B)**, and PFC group **(C)**; **(D)** lung injury scores; **(E)** Lung tissue W/D ratios; **(F)** Total cell count; **(G)** macrophage count; **(H)** neutrophil count; **(I)** lymphocyte count. Data are presented as means ± SEM. n = 6. * *p* < 0.05, ** *p* < 0.01, *** *p* < 0.001, **** *p* < 0.0001. Scale bar: 100 μm. Abbreviations: SW, seawater; PFC, perfluorocarbon; BALF, bronchoalveolar lavage fluid.

The pathological assessment of the SW group (Figure 2A) exhibited alveolar destruction, edema, hemorrhages, the extensive infiltration of inflammatory cells, and alveolar collapse (Figure 2B), and the lung injury scores (Figure 2D) were significantly increased compared to those for the control group. In the PFC group, the lung injuries, including alveolar destruction, edema, and hemorrhages, were alleviated (Figure 2C), and the lung injury scores were significantly reduced (Figure 2D) compared with those for the SW group.

Additionally, we performed total cell counting and differential cell counting using the H&E staining of BALF cells. Compared with the control group, the SW group exhibited a significantly elevated total cell count and higher macrophage, neutrophil and lymphocyte counts, while PFC inhalation effectively reversed the infiltration of total cells, neutrophils and lymphocytes induced by seawater (*p* < 0.05) (Figures 2F–I).

Then, we used the W/D ratios to evaluate the severity of the lung edema. The W/D ratios of the SW group were significantly increased compared with the control group (*p* < 0.001), while PFC administration downregulated the W/D ratios dramatically (*p* < 0.05) (Figure 2E).

### PFCs improved barrier injuries induced by seawater

**FIGURE 3:**
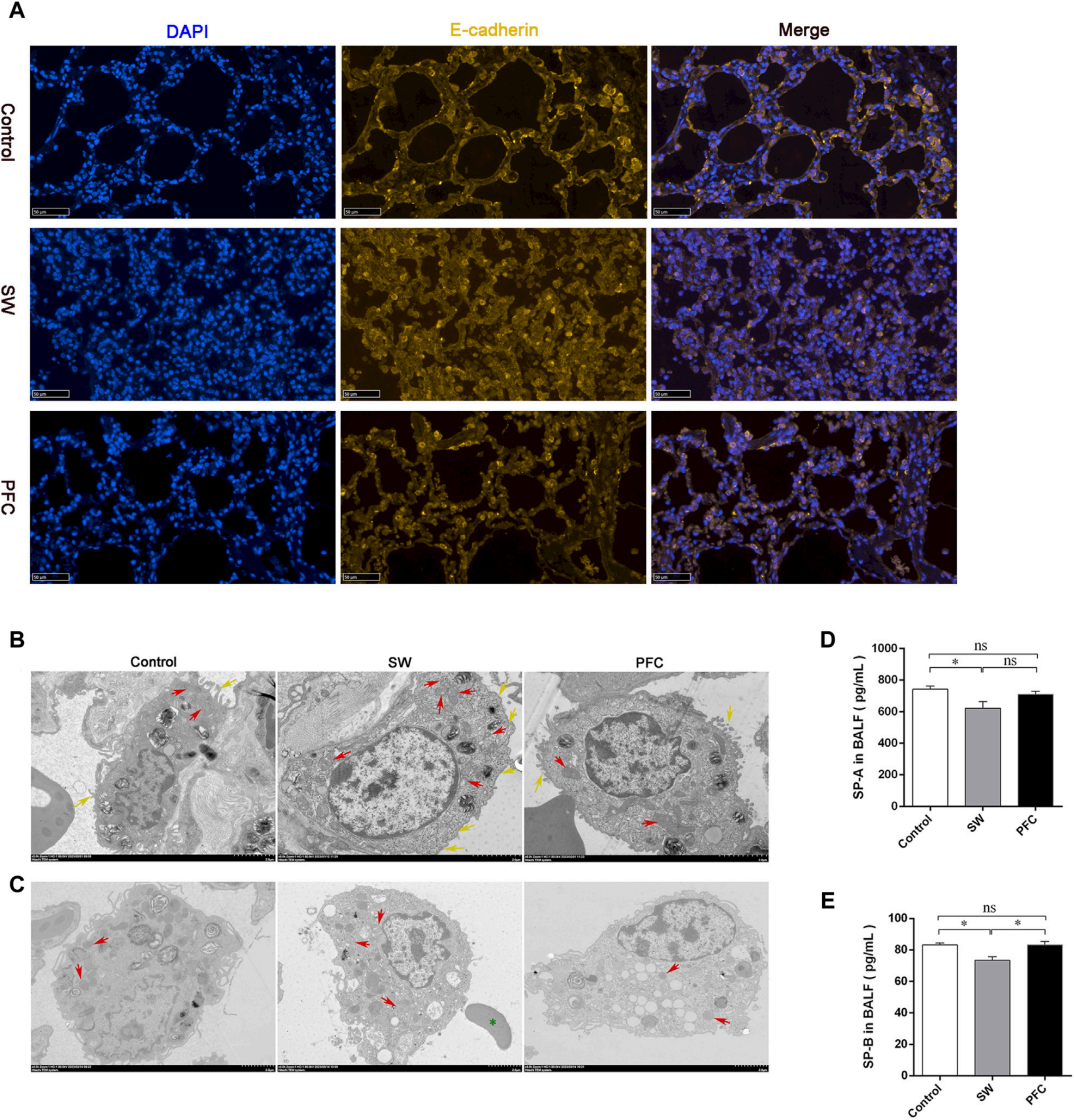
PFCs improved lung barrier injury and mitochondria morphological alterations induced by seawater. **(A)** The lung tissue is presented by immunofluorescence. Blue: nuclear staining (DAPI); yellow: E-cadherin. Scale bar: 50 μm. **(B)** Mitochondria TEM images of type II alveolar epithelial cells after treatment with seawater and seawater + PFC. Red arrows: swollen and damaged morphological mitochondria; yellow arrows: microvilli. Scale bar: 2 μm. **(C)** Mitochondria TEM images of macrophages after treatment with seawater and seawater + PFC. Red arrows: swollen and damaged morphological mitochondria; green asterisks: red blood cell. Scale bar: 2 μm. **(D)** SP-A protein levels in BALF; **(E)** SP-B protein levels in BALF. Data are presented as mean ± SEM. n = 6. ^∗^ *p* < 0.05. Abbreviations: SW, seawater; PFC, Perfluorocarbon; DAPI, 4′,6-diamidino-2′-phenylindole; TEM, transmission electron microscopy; SP-A, pulmonary surfactant A; SP-B, pulmonary surfactant B; BALF, bronchoalveolar lavage fluid.

E-cadherin is an adherens junctional protein with functions in the regulation of the epithelial architecture, regulating the paracellular permeability. As shown by the representative immunofluorescence images of E-cadherin in Figure 3A, seawater induced obvious epithelial swelling, structural rupture, and thickening, in contrast to the control group, while PFC treatment tended to relieve the aforementioned changes.

### PFCs improved type II alveolar epithelial (AT-Ⅱ) and mitochondria damages induced by seawater

Ultrastructural analysis was performed using TEM. AT Ⅱ cells from the SW group showed less microvilli (yellow arrows) and obvious swollen, fragmented mitochondria with disrupted cristae (red arrows), and PFC treatment alleviated the AT Ⅱ injury, as shown in Figure 3B. Macrophages, one of the most important immune cells in respiratory system, showed damaged morphological mitochondria (red arrows), and red blood cells (green asterisks) infiltrated the alveoli in the SW group, while PFCs reversed the damage induced by seawater (Figure 3C).

Moreover, to elucidate the function of AT Ⅱ, we performed ELISA to reveal the SP-A and SP-B concentrations in the BALF. Seawater induced a significant SP-A and SP-B deficiency, but PFCs increased the SP-B levels in the BALF dramatically (*p* < 0.05) (Figures 3D, E).

### PFCs upregulated HO-1 and NRF1 and downregulated NLRP3 inflammasome levels

**FIGURE 4:**
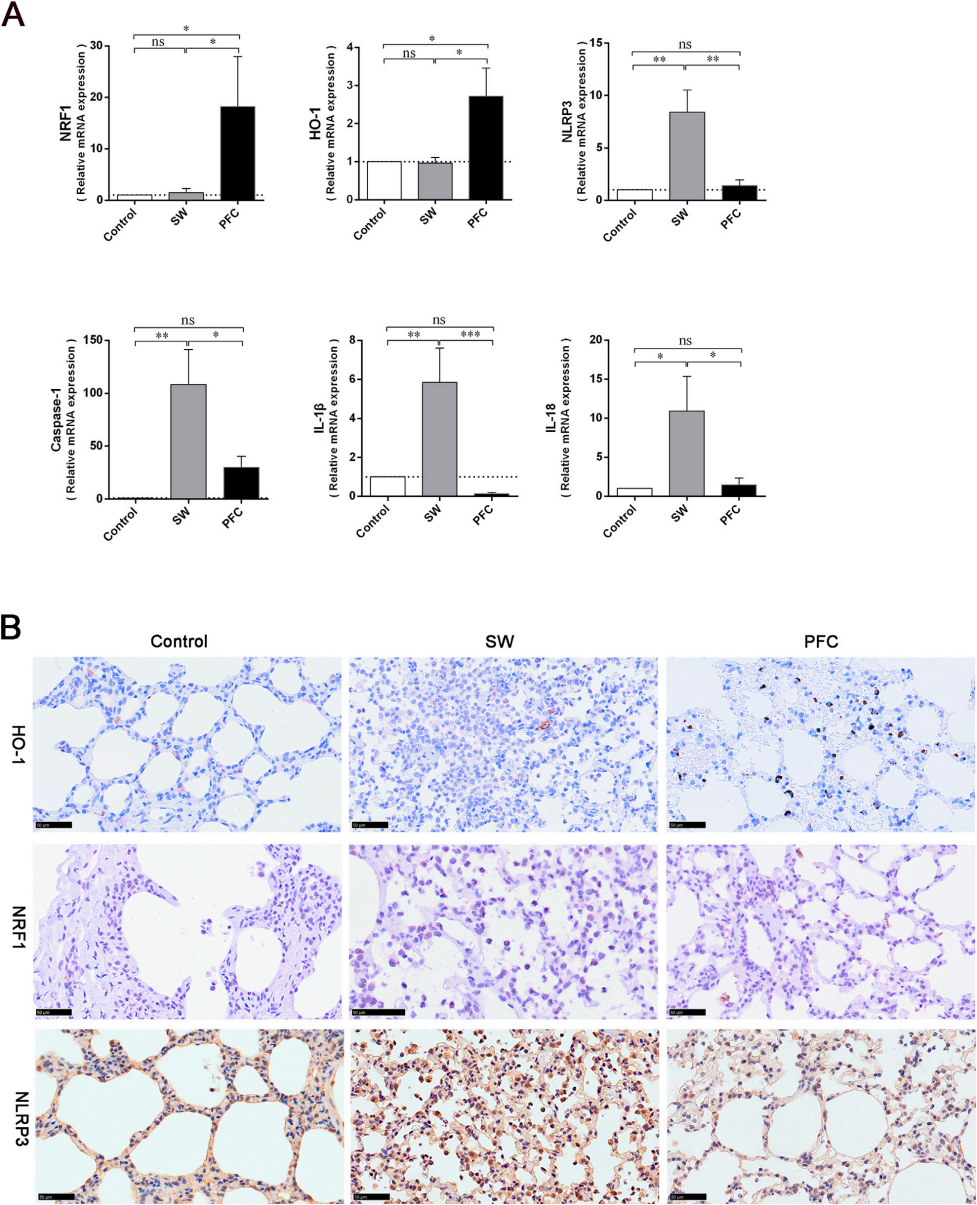
PFCs upregulated HO-1 and NRF1, and downregulated NLRP3 inflammasome expression in lung tissue. **(A)** The results obtained from qRT-PCR for NRF1, HO-1, NLRP3 inflammasomes, caspase-1, IL-1β, and IL-18. **(B)** Immunohistochemistry staining showing HO-1-positive cells, NRF1-positive cells and NLRP3-positive cells in lung tissue. Scale bar: 50 μm. Data are presented as mean ± SEM. n = 6. ^∗^ *p* < 0.05, ^∗∗^ *p* < 0.01, ^∗∗∗^ *p* < 0.001. Abbreviations: SW, seawater; PFC, perfluorocarbon; NRF1, nuclear respiratory factor-1; HO-1, heme oxygenase-1; NLRP3, NOD-like-receptor family pyrin domain-containing-3; IL-1β, interleukin-1β; IL-18, interleukin-18.

We employed qRT-PCR to quantify the mRNA expression levels of NRF1, HO-1, and NLRP3 inflammasomes. The PFC treatment increased the NRF1 and HO-1 expression levels and decreased NLRP3 expression levels compared with those in the control group and SW group (*p* < 0.05). Furthermore, the mRNA expression levels of caspase-1, IL-1β, and IL-18 were dramatically elevated after a seawater challenge, while PFC treatment significantly reversed these changes (*p* < 0.05) (Figure 4A). The results suggest that PFCs downregulated the NLRP3 inflammasomes as well as their downstream molecules and upregulated HO-1 and NRF1.

To further explore the mechanisms underlying PFCs’ therapeutic effects, we performed immunohistochemistry staining to detect the expression of HO-1, NRF1 and NLRP3 inflammasomes in lung tissues. We observed fewer HO-1-positive cells and NRF1-positive cells and more NLRP3 inflammasome-positive cells in the lung tissues of the SW group, while PFC treatment significantly reversed the HO-1, NRF1 and NLRP3 inflammasome expression induced by seawater (Figure 4B).

## Discussion

**FIGURE 5:**
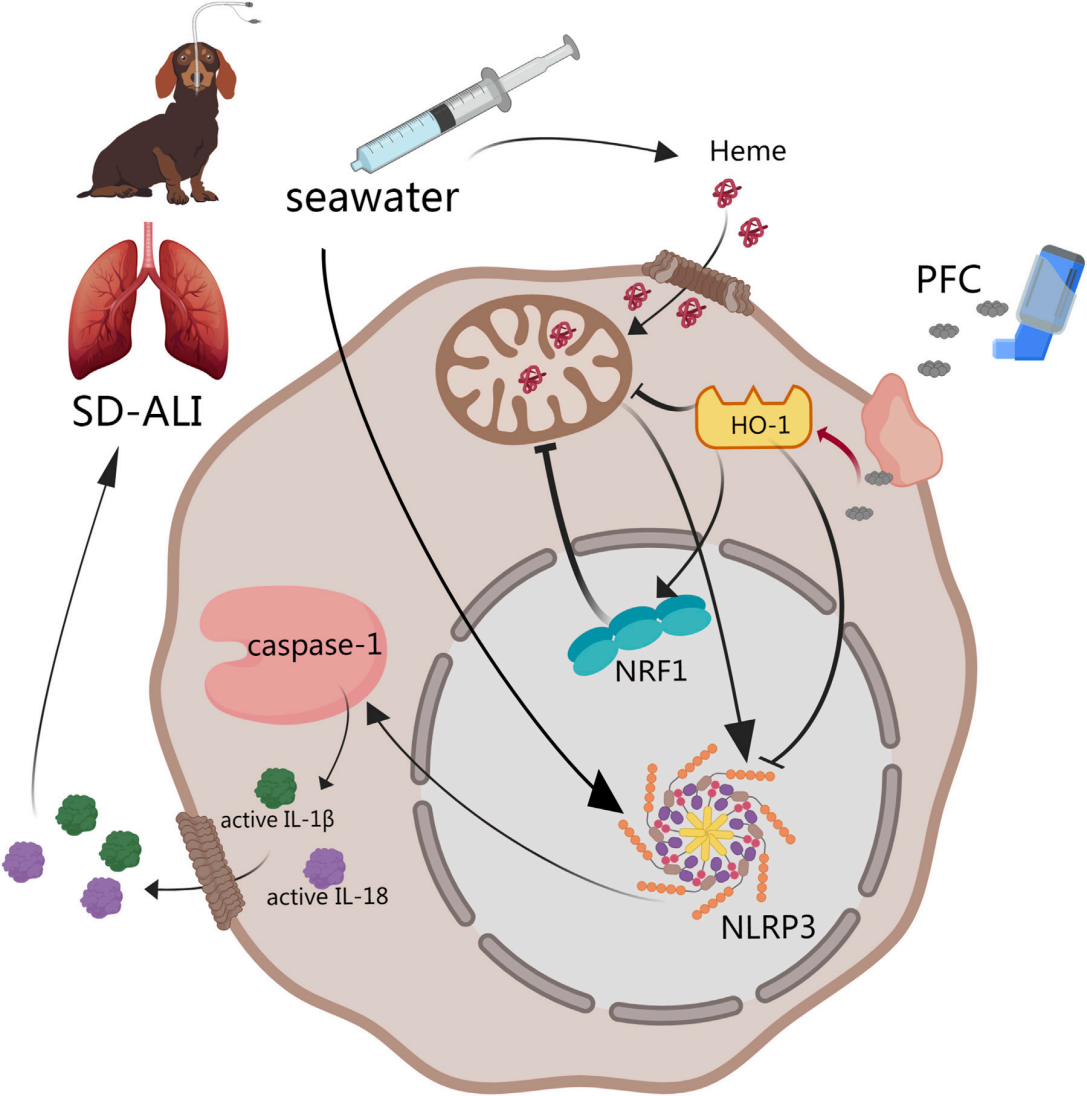
Abstract of our work. PFCs could downregulate NLRP3 inflammasome activation by the HO-1/NRF1 pathway and then decrease the release of mature IL-1β and IL-18 to alleviate acute lung injury induced by seawater. ① HO-1 may directly downregulate NLRP3 inflammasome gene expression. ② NRF1 may deactivate NLRP3 inflammasomes through mitochondrial quality control. Black line: pathway described in previous study. Red line: results obtained from our study. Abbreviations: SD-ALI, seawater-drowning-induced acute lung injury; PFC, perfluorocarbon; NRF1, nuclear respiratory factor-1; HO-1, heme oxygenase-1; NLRP3, NOD-like-receptor family pyrin domain-containing-3; IL-1β, interleukin-1β; IL-18, interleukin-18.

Drowning is one of the three major causes of death from unintentional injury in the world [26]. The lack of effective treatments is the main cause of death from drowning [2]. Seeking new drugs and methods to treat SD-ALI is important. Previous studies found that PFCs were effective in improving oxygenation in animals with lung injury and hypoxia, with the therapeutical effect mainly being ascribed to anti-inflammation and anti-oxidative stress effect by NF-κB pathway [18, 27, 28]. Our study established a SD-ALI model that fulfilled the standard for acute lung injury models updated in 2021 by the American Thoracic Society (ATS) [29], and it was the first time that dogs were used as SD-ALI model animals. The physiological structure characteristics and organ sizes of dogs are similar to those of humans, including the pulmonary, cardiovascular and cerebrovascular, and visual organs, which means they are suitable for the research and application of physical interventional therapy and intervention. The SD-ALI model showed significant pathological damage, a high W/D ratio, barrier damage and inflammatory cell infiltration. The results of our study showed that treatment with PFCs could ameliorate seawater-drowning-induced lung edema, barrier damage and inflammatory infiltration. The protective mechanism may involve the inhibition of NLRP3 activation via the HO-1/NRF1 pathway (Figure 5). To the best of our knowledge, this was the first time the effect of PFCs had been attributed to NLRP3 deactivation.

The blood–gas barrier, also known as the alveolar–capillary barrier, is the key functional element of the lung, serving as the site of oxygen and carbon dioxide exchange between the distal airspaces and the pulmonary vasculature [30]. West et al. previously described the stress failure of the blood–gas barrier in a rabbit lung using evidence from electron microscopy, with the concomitant translocation of red blood cells into the interstitium and/or airspaces [31]. The destruction of the blood–gas barrier could induce a perturbation in the fluid balance and result in pulmonary edema or acute lung injury [32]. The infection of alveolar epithelial cells and endothelial cells with severe acute respiratory syndrome coronavirus 2 (SARS-CoV-2) triggers an inflammatory response at the blood–gas barrier, inducing the release of IL-1β, interleukin-6 (IL-6), and tumor necrosis factor-α (TGF-α), leading to the disintegration and thickening of the blood–gas barrier [33]. We established SD-ALI canine models with hypoxemia, hypercapnia, and acidism, which indicated the disfunction of the blood–gas barrier. PFCs were administrated by inhalation, which can work immediately and directly on the lung. We then observed morphological changes in the blood–gas barrier by the immunofluorescence staining of lung tissue, characterized as epithelial swelling, structural rupture, and thickening. Our work demonstrated that PFC administration significantly improved the lung barrier structure and oxygenation. Moreover, PFCs could reduce injury to AT Ⅱ cells, which produce and release pulmonary surfactant to maintain alveolar surface tension.

Our study attempted to visualize the structure of the pulmonary alveoli using pCLE in a living body. The pCLE technique uses a laser wavelength that mainly allows for the visualization of elastic fibers in lung tissue. Many studies have been conducted to assess the applicability of pCLE in central neoplasms, diffuse parenchymal lung disease, lung emphysema, pneumonia, acute lung injury, etc. [34–38]. Our study visualized the changes in images that occurred under acute lung injury induced by seawater drowning in canine models, characterized by an increased density of elastic structures, and large drops of intra-alveolar secretions compared with the control group and PFC group, which were also supported by quantitative analysis using the VAI. The VAI may help us to assess the air content of lung tissue. To the best of our knowledge, this is the first pCLE image description and quantitative analysis of acute lung injury induced by seawater drowning.

Inflammatory responses are important mechanisms of SD-ALI. We found substantial inflammatory infiltrations in lung tissues from the SW group. Previous studies have found that seawater can increase the production of pro-inflammatory cytokines, including IL-6, interleukin-10 (IL-10), IL-1β, and TNF-α, *in vivo* and *in vitro* [39–41]. We found that seawater challenge upregulated IL-1β and IL-18. Moreover, the vaporization of PFCs could alleviate inflammatory cell infiltration and reduce the expression of IL-1β and IL-18 in lung tissue. NLRP3 inflammasomes are cytoplasmic high-molecular-weight protein platforms of caspase-1 activation in response to microbial invasion and damage signals and have been found to be activated in macrophages and AT Ⅱ cells by a wide spectrum of stimuli, including seawater [42]. The formation of these protein complexes activates caspase-1, which is involved in the maturation of the proinflammatory cytokines IL-1β and IL-18 into their biologically active forms [42]. The regulation of the NLRP3 inflammasome activation state is a promising target for neuronal necroptosis, osteoarthritis, and respiratory system diseases [43, 44]. Based on the above, we hypothesized that the therapeutic effect of PFCs may be achieved through NLRP3 inflammasomes. To elucidate whether PFCs influence the seawater-induced activation of NLRP3 inflammasomes, we confirmed the seawater-induced activation of NLRP3 inflammasomes in lung tissue based on their mRNA and protein levels, which were consistent with previous studies [21]. Then, we found that PFC administration significantly deactivated NLRP3 inflammasomes, which was verified by means of qRT-PCR and immunohistochemistry. Moreover, PFC vaporization also downregulated caspase-1, a signal factor downstream of NLRP3 and the key molecule linking NLRP3 inflammasome activation and IL-1β and IL-18 maturation.

Knowing that NLRP3 inflammasome activation is regulated by multiple complicated positive and/or negative regulatory pathways providing upstream signals [42, 45], we attempted to find pathways involved in this activity. HO-1 is a rate-limiting enzyme of heme degradation that catalyzes the degradation of free heme to biliverdin, ferrous iron, and carbon monoxide [46]. HO-1 is reported to have antioxidant and anti-inflammatory effects in acute lung injury, and also can decrease NLRP3 inflammasome deactivation induced by heme [39, 43, 44, 47, 48]. NRF1 is a phosphorylated nuclear protein that functions as a transcription factor, activating the expression of key metabolic genes regulating cellular growth and nuclear genes required for respiration, heme biosynthesis, and mitochondrial DNA transcription and replication [49]. However, the function of NRF1 has not been fully elucidated. HO-1 regulates mitochondrial biogenesis and activates the redox-sensitive transcriptional program for mitochondrial quality control through NRF1. This pathway is implicated in the mitigation of various injuries, including organ fibrosis, ischemic and drug-induced cardiomyopathy, acute lung injury, and ARDS [48, 50]. A previous study reported that mitochondrial damage and dysfunction could promote epithelial barrier damage through calcium dysregulation, energy failure, apoptosis, and the loss of heme homeostasis [51, 52]. Our work observed mitochondrial damage in the SW group via TEM, characterized by obviously swollen, fragmented mitochondria with disrupted cristae, suggesting that the HO-1/NRF1 pathway may participate in mitochondrial quality control during the pathophysiological process of SD-ALI. NRF1 may directly downregulate NLRP3 inflammasome gene expression within the nucleus or promote mitochondrial quality control to influence heme metabolism. The elaboration of the HO-1/NRF1 pathway to NLRP3 inflammasome activation needs further investigation.

In summary, we provided evidence that the inhalation of vaporized PFCs suppressed SD-ALI in canine models, alleviated hypoxemia and blood–gas barrier injuries, and reduced inflammatory infiltration and cytokine release. The mechanisms of PFCs’ therapeutic effects may be attributed to the regulation of NLRP3 inflammasomes’ activation via the HO-1/NRF1 pathway. This study offers a meaningful exploration of PFCs’ therapeutic mechanisms *in vivo*.

## Data Availability

The datasets presented in this article are not readily available because confidentiality period lasts for 10 years. Requests to access the datasets should be directed to suellen1988@163.com.
